# Association between immune-related adverse events and immunotherapy efficacy in non-small-cell lung cancer: a meta-analysis

**DOI:** 10.3389/fphar.2023.1190001

**Published:** 2023-05-22

**Authors:** Li Lin, Yu Liu, Chen Chen, Anhua Wei, Wei Li

**Affiliations:** ^1^ Department of Oncology, Wuhan Asia General Hospital, Wuhan, China; ^2^ Department of Pharmacy, Tongji Hospital, Tongji Medical College, Huazhong University of Science and Technology, Wuhan, China

**Keywords:** immune-related adverse events, clinical efficacy, immune checkpoint inhibitors, meta-analysis, non-small-cell lung cancer

## Abstract

**Objective:** Our study aimed to identify potential correlations between anti-tumor efficacy and immune-related adverse events (irAEs) in non-small-cell lung cancer (NSCLC).

**Methods:** We conducted a comprehensive search of online electronic databases up to March 2023 to identify any correlations between irAEs and immune checkpoint inhibitor (ICI) efficacy in NSCLC. We used meta-analysis RevMan 5.3 software to calculate pooled results.

**Results:** Our meta-analysis of 54 studies revealed that patients who experienced irAEs achieved a significantly higher objective response rate (*p* < 0.00001) and longer progression-free survival (PFS) (*p* < 0.00001) and overall survival (OS) (*p* < 0.00001) than those who did not experience irAEs. Additionally, patients with ≥2 irAEs had better PFS, whereas no significant difference was observed between patients with or without squamous cell carcinoma. Subgroup analysis of irAE types indicated that irAEs (thyroid dysfunction and gastrointestinal, skin, or endocrine irAEs) were associated with better PFS and OS. However, no significant differences were observed between patients with pneumonitis or hepatobiliary irAEs.

**Conclusion:** Our study showed that the occurrence of irAEs was a strong predictor of survival efficacy in patients with NSCLC treated with ICIs. Specifically, patients with ≥2 irAEs and those with thyroid dysfunction and gastrointestinal, skin, or endocrine irAEs achieved a better survival benefit.

**Systematic Review Registration:** Website: https://www.crd.york.ac.uk/prospero/, Identifier: CRD42023421690

## Introduction

Immunotherapy, which targets the programmed death-1 (PD-1) and programmed death-ligand 1 (PD-L1) pathway, has emerged as a revolutionary and highly efficient treatment alternative for advanced-stage non-small-cell lung cancer (NSCLC) (Assi et al., 2018). Specifically, immune checkpoint inhibitors (ICIs), including those that target the PD-1/PD-L1 axis, enhance T-cell-mediated attack and exert anti-tumor effects (Wei et al., 2018).

In contrast to conventional chemotherapy, the use of ICIs can sometimes lead to a unique toxicity effect resembling autoimmune disorders. This “self-response” of the immune system is known as immune-related adverse events (irAEs) (Young et al., 2018).

Several retrospective studies have highlighted that the occurrence of irAEs in patients with melanoma is associated with improved survival outcomes (Freeman-Keller et al., 2016; Hua et al., 2016; Nakamura et al., 2017), suggesting that irAEs may be a predictive marker for the clinical benefit of ICIs. However, achieving the maximum therapeutic efficacy with ICIs requires a careful balance between anti-tumor immunity and autoimmunity. Conversely, studies on patients with advanced NSCLC have identified a correlation between adverse events and clinical outcomes of ICIs. Whether the occurrence of irAEs is associated with the clinical efficacy in NSCLC remains a subject of ongoing research. However, the results of current studies investigating the correlation between anti-tumor efficacy and the development of irAEs have been inconsistent (Kothari et al., 2017; Haratani et al., 2018; Cortellini et al., 2019).

With the aim to provide a systematical, up-to-date assessment of the potential predictive value of irAEs in NSCLC and gain a better understanding of the relationship between irAEs and clinical outcomes, we performed an update meta-analysis of the association between the occurrence of irAEs and anti-tumor efficacy.

## Materials and methods

### Search strategy

The meta-analysis was based on the Cochrane Manual of Intervention System Assessments and Guidelines for Systematic Reviews and Meta-Analyses. PubMed, Embase, and the Cochrane Library were searched for articles published up to March 2023. The process was conducted to find all relevant studies using the following keywords: “non-small cell lung cancer” AND “immune checkpoint inhibitors” OR “immunotherapy” AND “immune-related adverse events” AND “prognosis” terms, and the relevant Medical Subject Heading terms were used during the literature search process. The reference lists were also checked for retrieving additional relevant articles.

### Eligibility criteria

Articles that met the following criteria were included: (1) patients had clinical diagnosis of NSCLC treated with an ICI; (2) trials focused on assessing the effectiveness of ICI in relation to the advent of irAEs; (3) outcomes of interest were effectiveness (overall survival [OS], progression-free survival [PFS], and tumor response) and selection of AE designation related to treatment; and (4) only full texts were included.

### Quality assessment

Two authors (Li Lin and Yu Liu) separately justified the quality of the retrieved articles. Study quality was assessed using the Newcastle–Ottawa Quality Assessment Scale (Cook and Reed, 2015). The process was conducted by two researchers independently, and differences were resolved through discussion. The Newcastle–Ottawa Scale method uses three domains to assess the quality of cohort studies: the selection of patients with cancer, comparability between two groups, and assessment of outcomes. According to the NOS method, four, two, and three points were awarded to the three domains, respectively. Studies with ≥7 points were identified as having high quality, but those with ≤6 points were identified as having low quality. Publication bias was evaluated using funnel plots.

### Data extraction

A data extraction form was used independently to retrieve the content containing the first author, publication year, ICI treatment regimen, region, number of patients, mean age, study design, follow-up period, and outcomes of interest. Two researchers (Li Lin and Yu Liu) independently evaluated the data. If there was a disagreement, a third researcher (Wei Li) resolved the disagreement through discussion.

### Statistical analysis

Heterogeneity between studies was analyzed using the I^2^ statistic (Higgins and Thompson, 2002). A value of I^2^ > 50% implied a high degree of heterogeneity (Higgins et al., 2003). The random-effects model was used when there was high heterogeneity among the articles; otherwise, a fixed-effects model was used. Statistically significant differences were identified using a *p*-value of <0.05. Statistical analyses were conducted using the ReviewManager software package version 5.3 (RevMan; Cochrane Collaboration, Oxford, United Kingdom). Odds ratios (ORs) and 95% confidence intervals (CIs) were used for binary data and effect sizes in the meta-analysis. Forest plots were used to present the results of the study.

## Results

### Overview of the literature search

**FIGURE 1:**
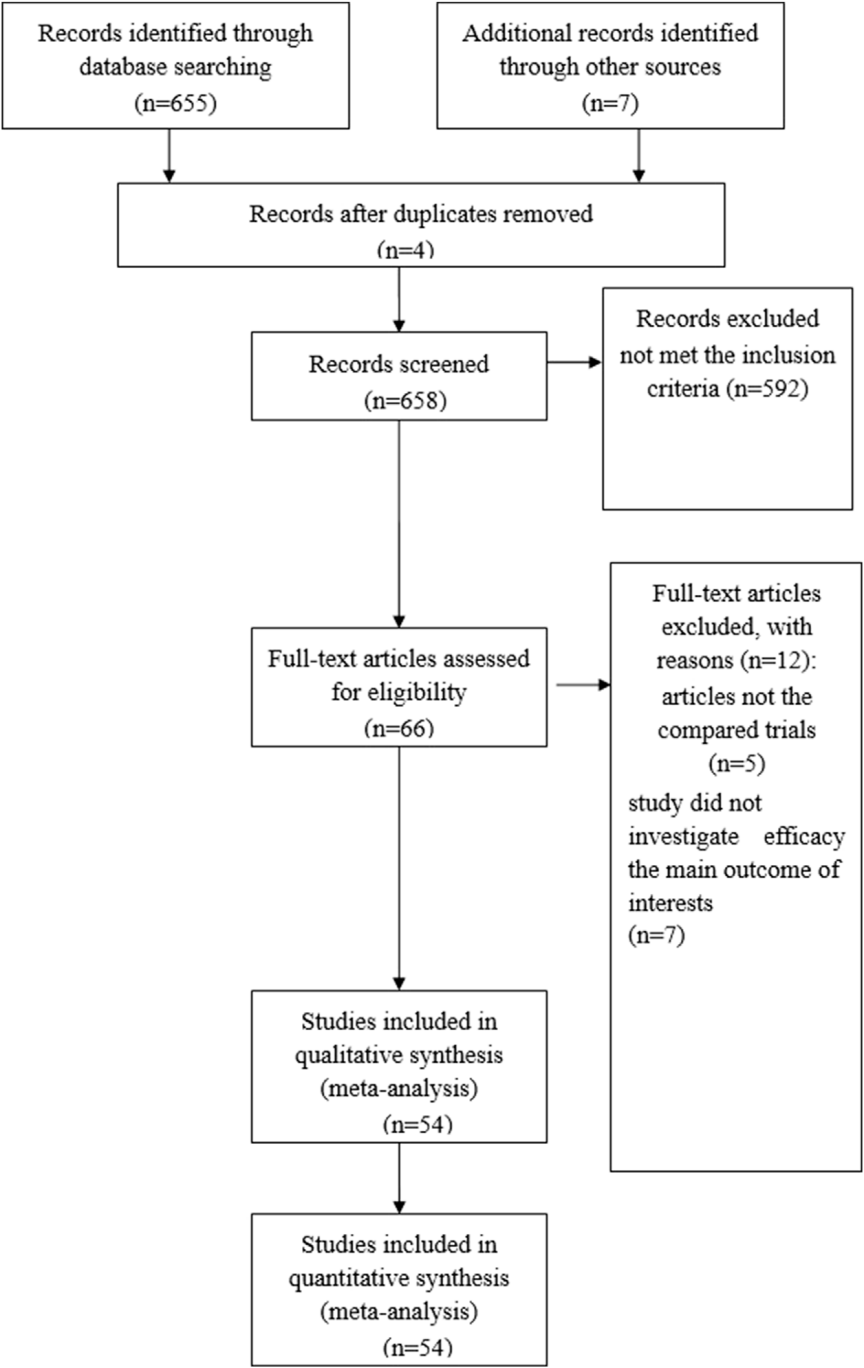
PRISMA flow chart of the selection process to identify studies eligible for pooling.

A total of 658 articles were identified as potentially eligible for inclusion. Using the criteria outlined in the Methods section, 66 publications were evaluated by browsing the full set of studies; however, some did not provide sufficient outcome data from the two approaches. Finally, 54 articles (Ahn et al., 2019; Akamatsu et al., 2020; Aso et al., 2020; Baldini et al., 2020; Berner et al., 2019; Bjørnhart et al., 2019; Bouhlel et al., 2020; Campredon et al., 2019; Cortellini et al., 2019; Chen et al., 2021; Chmielewska et al., 2021; Cui et al., 2020; Cortijo-Cascajares et al., 2023; Daniello et al., 2021; Dupont et al., 2019; Fujimoto et al., 2018; Fukihara et al., 2019; Grangeon et al., 2019; Guezour et al., 2022; Haratani et al., 2018; Imai et al., 2020; Isono et al., 2021; Kim et al., 2017; Kobayashi et al., 2020; Ksienski et al., 2019; Kubo et al., 2020; Lesueur et al., 2018; Lisberg et al., 2018; Morimoto et al., 2021; Naqash et al., 2020; Noguchi et al., 2020; Osorio et al., 2017; Owen et al., 2018; Park et al., 2021; Peiró et al., 2019; Ricciuti et al., 2019; Rose et al., 2021; Rubino et al., 2021; Sato et al., 2018; Sayer et al., 2023; Schweizer et al., 2020; Shukla et al., 2021; Sonehara et al., 2021; Socinski et al., 2023; Sugano et al., 2020; Suh et al., 2018; Tambo et al., 2020; Teraoka et al., 2017; Thuillier et al., 2021; Toi et al., 2018; Wang et al., 2022; Yamaguchi et al., 2020; Zhang et al., 2021; Zhou et al., 2021) were assessed for eligibility in the meta-analysis. Figure 1 illustrates the search process. A brief description of the 54 studies is provided in Table 1.

**TABLE 1:** Characteristics of the include studies.

Study year	Study design	Immune checkpoint inhibitor	No. of patients (male/female)	Median age	Region	Follow-up period	NOS
Haratani et al. (2018)	Retrospective study	Nivolumab	134 (90/44)	68	Japan	December 2015 and August 2016	7
Sato et al. (2018)	Retrospective study	Nivolumab	38 (28/10)	68.5	Japan	December 2015 and February 2017	8
Teraoka et al. (2017)	Retrospective study	Nivolumab	43 (27/16)	70	Japan	January and December 2016	7
Lesueur et al. (2018)	Retrospective study	Nivolumab	104 (67/37)	60.3	France	During 2014	7
Owen et al. (2018)	Retrospective study	Nivolumab	91 (39/52)	67	America	September 2014 to June 2016	7
Toi et al. (2018)	Retrospective study	Nivolumab	137 (61/76)	68	Japan	January 2016 and January 2018	7
Baldini et al. (2020)	Retrospective study	Nivolumab	1,959 (1327/632)	/	Italy	/	7
Ricciuti et al. (2019)	Retrospective study	Nivolumab	195 (128/67)	63	Italy	October 2013 and September 2017	8
Naqash et al. (2020)	Retrospective study	Nivolumab	531 (NA)	/	America	/	7
Lisberg et al. (2018)	Retrospective study	Pembrolizumab	97 (50/47)	67	America	August 2012 and December 2016	8
Osorio et al. (2017)	Retrospective study	Pembrolizumab	51 (NA)	/	America	4 March 2011 and 11 December 2018	7
Tambo et al. (2020)	Retrospective study	Pembrolizumab	95 (NA)	/	Japan	February 2017 to December 2018	7
Kim et al. (2017)	Retrospective study	Nivolumab/pembrolizumab	58 (43/15)	63.1	Korea	89.0 days	8
Ksienski et al. (2019)	Retrospective study	Nivolumab/pembrolizumab	271 (116/114)	66	Canada	June 2015 to November 2017	8
Cortellini et al. (2019)	Retrospective study	Nivolumab/pembrolizumab	559 (379/180)	/	Italy	/	7
Suh et al. (2018)	Retrospective study	Nivolumab/pembrolizumab	54 (42/12)	70	Korea	/	7
Yamaguchi et al. (2020)	Retrospective study	Nivolumab/pembrolizumab	131 (98/33)	77	Japan	January 2016 and February 2018	7
Grangeon et al. (2019)	Retrospective study	Anti-PD-L1 or anti-PD-1	270 (NA)	/	France	April 2013 to February 2017	8
Aso et al. (2020)	Retrospective study	Nivolumab/pembrolizumab	155 (117/38)	68	Japan	January 2016 to April 2018	7
Sonehara et al. (2021)	Retrospective study	Nivolumab/pembrolizumab/atezolizumab	80 (65/15)	/	Japan	February 2016 and February 2020.	7
Morimoto et al. (2021)	Retrospective study	Pembrolizumab/atezolizumab	70 (51/19)	69.5	Japan	January 2019 and September 2019	7
Shukla et al. (2021)	Retrospective study	Pembrolizumab	92 (59/33)	64	Japan	March 2015 to November 2016	8
Chmielewska et al. (2021)	Retrospective study	Nivolumab	35 (20/15)	65.8	Poland	November 2016 to January 2020	8
Daniello et al. (2021)	Retrospective study	Nivolumab/pembrolizumab/atezolizumab	894 (536/358)	65	Germany.	October 2012 and June 2020	8
Chen et al. (2021)	Retrospective study	Anti-PD-L1 or anti-PD-1	191 (139/52)	/	China	August 2016 to November 2019	7
Fujimoto et al. (2018)	Retrospective study	Nivolumab	613 (NA)	/	Japan	January and December 2016	6
Ahn et al. (2019)	Retrospective study	Nivolumab or pembrolizumab	155 (113/42)	64	Korea	March 2014 and January 2019	7
Berner et al. (2019)	Retrospective study	Anti-PD-1	73 (44/29)	68.1	Switzerland	1 July 2016 to 31 December 2018	7
Bjørnhart et al. (2019)	Retrospective study	Anti-PD-L1 or anti PD-1	118 (44/74)	66	Denmark	September 2015 to April 2018	7
Imai et al. (2020)	Retrospective study	Pembrolizumab	87 (40/47)	79	Japan	February 2017 and February 2018	7
Sugano et al. (2020)	Retrospective study	Nivolumab, pembrolizumab, or atezolizumab	130 (98/32)	/	Japan	December 2015 and November 2018	7
Noguchi et al. (2020)	Retrospective study	Pembrolizumab	94 (82/12)	/	Japan	February 2017 to August 2019	7
Kubo et al. (2020)	Retrospective study	Nivolumab or pembrolizumab	95 (77/18)	79	Japan	December 2015 to December 2017	7
Akamatsu et al. (2020)	Retrospective study	Nivolumab, pembrolizumab, or atezolizumab	23 (18/5)	69	Japan	December 2015 and September 2018	7
Campredon et al. (2019)	Retrospective study	Nivolumab	183 (72/111)	61	France	May 2015 and December 2016	7
Cui et al. (2020)	Retrospective study	Anti-PD-L1 or anti-PD-1	276 (205/71)	61	China	September 2015 and 31 August 2019	7
Dupont et al. (2019)	Retrospective study	Nivolumab	191 (121/70)	63	France	19 September 2014 and 31 December 2016	7
Fukihara et al. (2019)	Retrospective study	Nivolumab or pembrolizumab	170 (125/45)	/	Japan	January 2016 and March 2018	8
Isono et al. (2021)	Retrospective study	Nivolumab, pembrolizumab, or atezolizumab	180 (140/40)	68.5	Japan	1 January 2016 to 31 March 2019	7
Kobayashi et al. (2020)	Retrospective study	Ipilimumab, nivolumab, pembrolizumab, or atezolizumab	108 (79/29)	67	Japan	2 November 2015 and 30 August 2019	7
Bouhlel et al. (2020)	Retrospective study	Nivolumab	69 (NA)	/	France	March 2015 to March 2017	7
Peiró et al. (2019)	Retrospective study	Nivolumab	55 (NA)	/	Spain	During 2016	7
Park et al. (2021)	Retrospective study	Pembrolizumab or nivolumab	1,181 (932/249)	67	Korea	During 2019	7
Rose et al. (2020)	Retrospective study	Ipilimumab, nivolumab, pembrolizumab, or atezolizumab	233 (NA)	70	America	July 2011 and October 2019	7
Rubino et al. (2021)	Retrospective study	Pembrolizumab or nivolumab	251 (109/64)	70.6	Italy	1 October 2017 to 31 July 2020	8
Thuillier et al. (2021)	Retrospective study	Nivolumab	134 (94/40)	62.5	France	20 July 2015 and 30 June 2018	7
Zhang et al. (2021)	Retrospective study	Pembrolizumab	63 (32/31)	65.5	America	January 2014 and February 2019	7
Schweizer et al. (2020)	Retrospective study	Anti-PD-L1	104 (76/28)	/	Germany	/	7
Zhou et al. (2021)	Retrospective study	Anti-PD-1	191 (138/53)	/	China	10 October 2016 and 1 April 2020	7
Sayer et al. (2023)	Retrospective study	Anti-PD-L1 or anti-PD-1	354 (190/164)	/	America	2014 and 2018	8
Socinski et al. (2023)	Retrospective study	Atezolizumab	2,503 (1519/984)	63.1	America	During February 2022	8
Guezour et al. (2022)	Retrospective study	Ipilimumab, nivolumab, or pembrolizumab	201 (132/69)	63	France	1 January 2016 and 31 December 2019	7
Cortijo-Cascajares et al. (2023)	Retrospective study	Nivolumab	75 (51/24)	/	Spain	February 2015 to May 2020	7
Wang et al. (2022)	Retrospective study	Anti-PD-L1 or anti-PD-1	222 (179/43)	/	China	/	7

irAEs, immune-related adverse events; NOS, Newcastle–Ottawa Quality Assessment Scale; NA, not available.

A total of 54 studies had high methodological quality. Table 1 summarizes the quality appraisal process.

### Outcomes and synthesis of results

#### Pooled analysis of the OS between IrAEs and ICI efficacy

**FIGURE 2:**
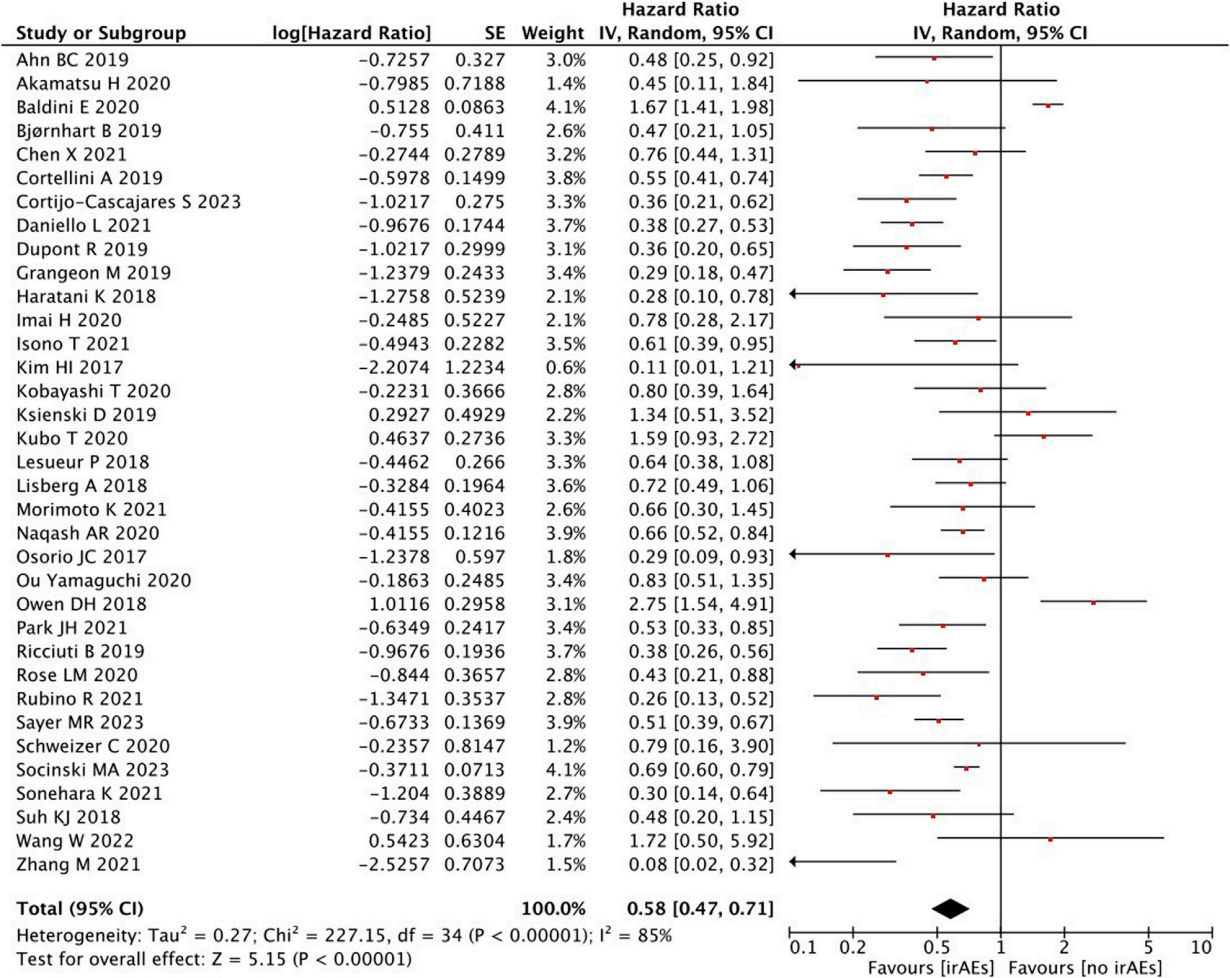
Analysis of OS between IrAEs and ICI efficacy.

A high statistical between-study heterogeneity was found in the ORs of the studies (I^2^ = 85%), and a random-effects model was used for merging. As shown in Figure 2, the pooled effect size estimates showed that there was a statistically significant difference in OS when comparing the irAEs with the no-irAE group (HR = 0.58, 95% CI = 0.47–0.71, *p* < 0.00001).

**FIGURE 3:**
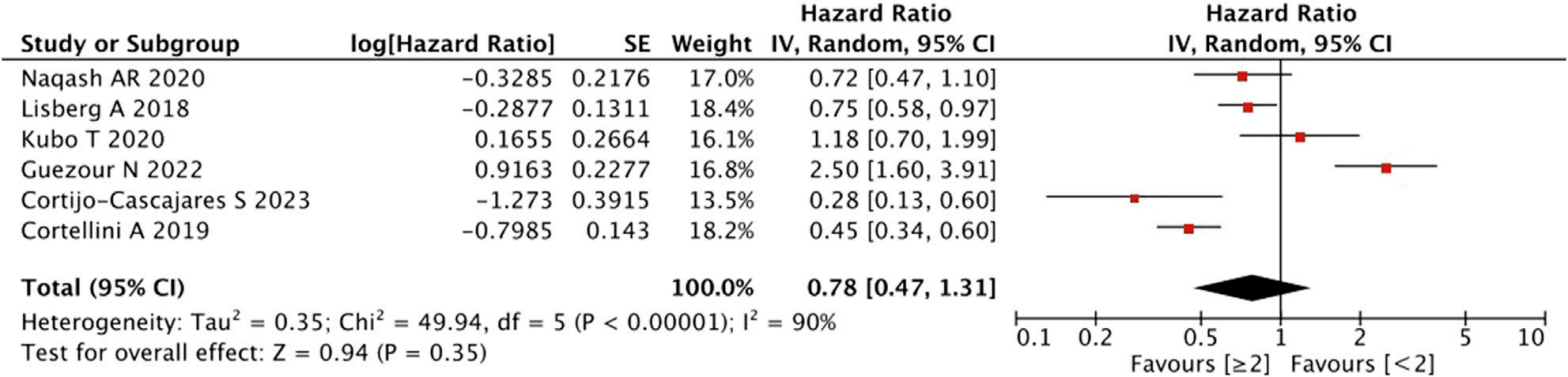
Sub-group analysis of OS between IrAEs and ICI efficacy with the number of irAEs.

With regard to the number of irAEs developed, no significant difference was found in patients with ≥2 irAEs than those with <2 irAEs (HR = 0.78, 95% CI = 0.47–1.31, *p* = 0.35) (Figure 3).

**FIGURE 4:**
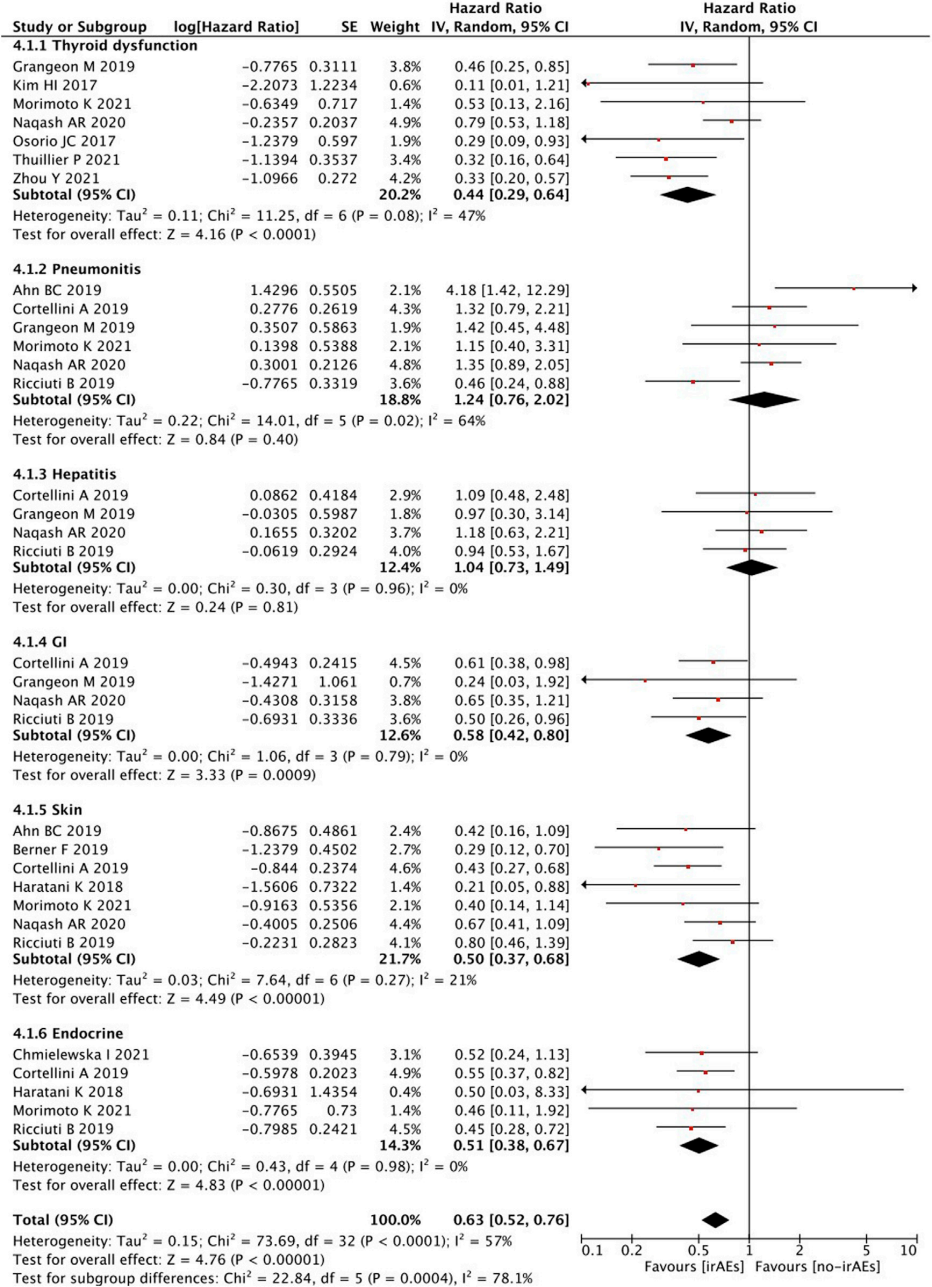
Sub-group analysis of OS between IrAEs and ICI efficacy with types of irAEs.

When survival outcomes were analyzed according to the types of irAEs, patients who developed any irAEs (*p* < 0.00001), thyroid dysfunction (*p* < 0.00001), and gastrointestinal (*p* = 0.0009), skin (*p* < 0.00001), or endocrine (*p* < 0.00001) irAEs experienced a significantly longer OS, whereas there was no significant change observed in patients with pneumonitis (*p* = 0.40) and hepatobiliary (*p* = 0.81) irAEs (Figure 4).

**FIGURE 5:**
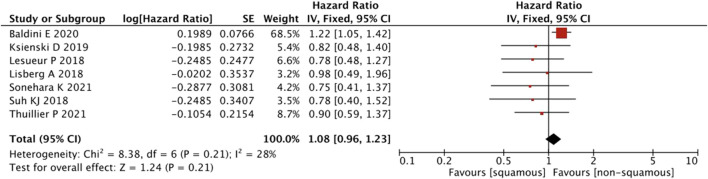
Sub-group analysis of OS between IrAEs and ICI efficacy with pathological subtypes of irAEs.

In terms of the pathological subtype, no differences were observed between the presence and absence of squamous cell carcinoma (HR = 1.08, 95% CI = 0.96–1.23, *p* = 0.21) (Figure 5).

#### Pooled analysis of the PFS between the IrAEs and ICI efficacy

**FIGURE 6:**
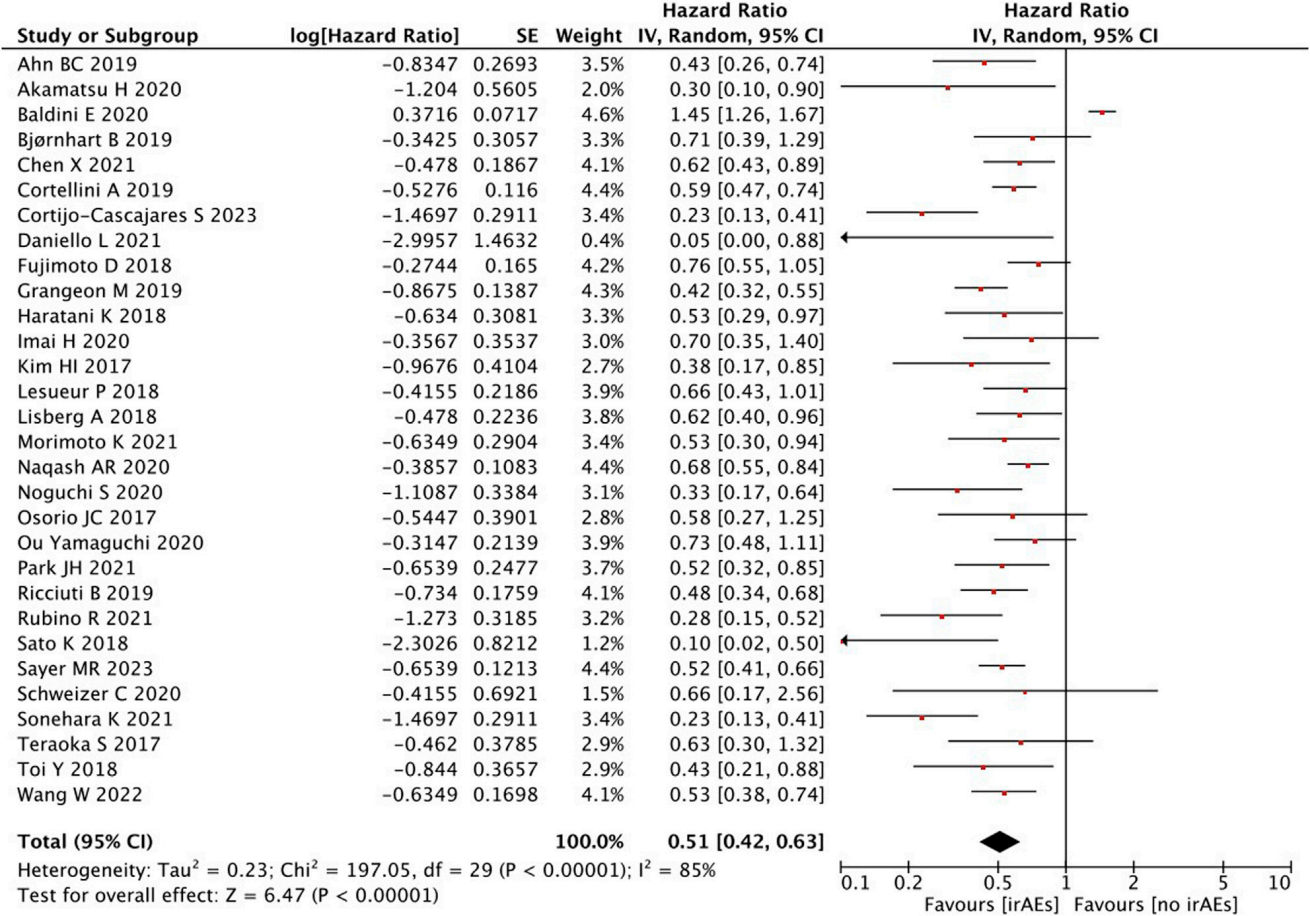
Analysis of PFS between IrAEs and ICI efficacy.

A statistical between-study heterogeneity was found in the OR of studies (I^2^ = 85%); therefore, a random-effects model was used for merging. When the PFS was pooled, it was found that patients with irAEs were associated with a better PFS than those without irAEs (HR = 0.51, 95% CI = 0.42–0.63, *p* < 0.00001) (Figure 6).

**FIGURE 7:**
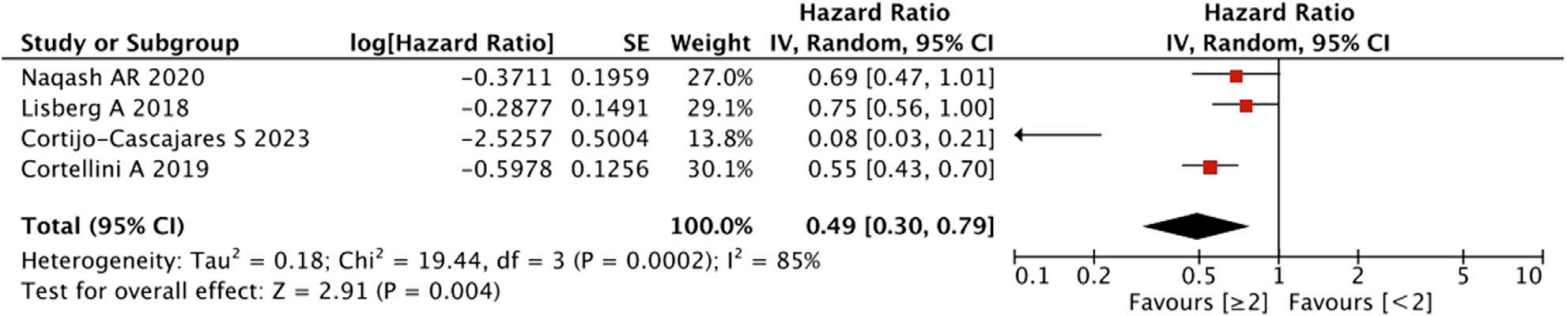
Sub-group analysis of PFS between IrAEs and ICI efficacy with the number of irAEs.

As shown in Figure 7, the pooled effect size estimates showed a significant statistical difference in PFS for patients with an increasing number of irAEs compared with those with <2 irAEs (HR = 0.49, 95% CI = 0.30–0.79, *p* = 0.004).

**FIGURE 8:**
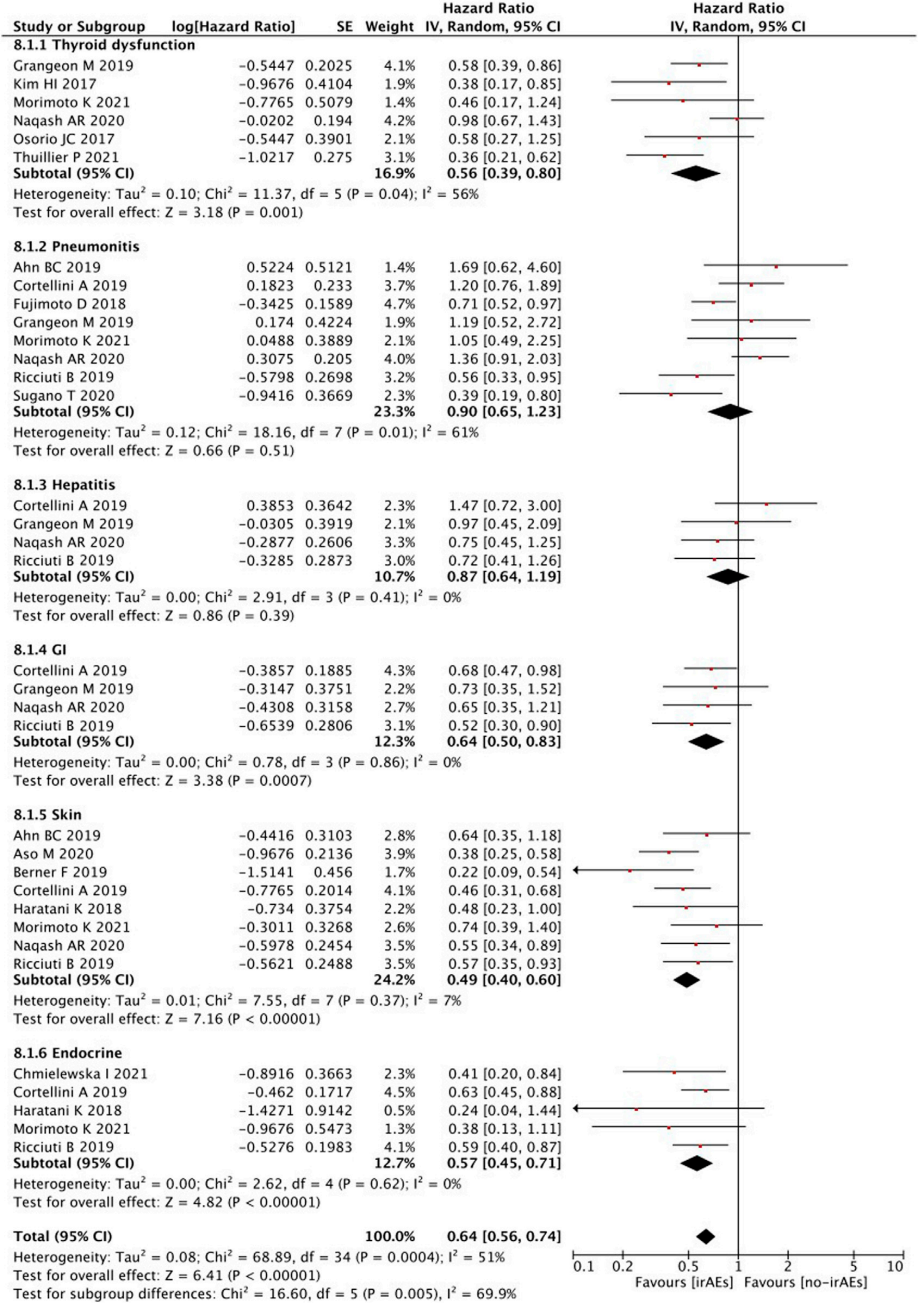
Sub-group analysis of PFS between IrAEs and ICI efficacy with types of irAEs.

Subgroup analyses of treatment-related AEs revealed that PFS was significantly better in patients with any irAEs (*p* < 0.00001), thyroid dysfunction (*p* = 0.001), and gastrointestinal (*p* = 0.0007), skin (*p* < 0.00001), or endocrine (*p* < 0.0001) irAEs (Figure 8). However, no statistically significant differences were detected in the PFS of patients with irAEs associated with pneumonitis (*p* = 0.51) or hepatobiliary disease (*p* = 0.39).

**FIGURE 9:**
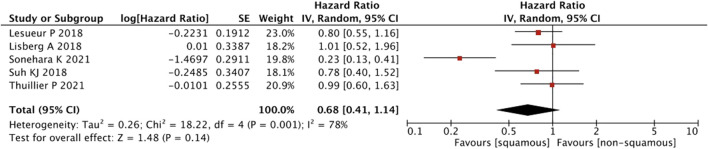
Sub-group analysis of PFS between IrAEs and ICI efficacy with pathological subtypes.

As shown in Figure 9, a pooled estimates of effect size showed no significant statistical difference in patients with or without squamous cell carcinoma (HR = 0.68, 95% CI = 0.41–1.14, *p* = 0.14).

#### Pooled analysis of the objective response rate between IrAEs and ICI efficacy

**FIGURE 10:**
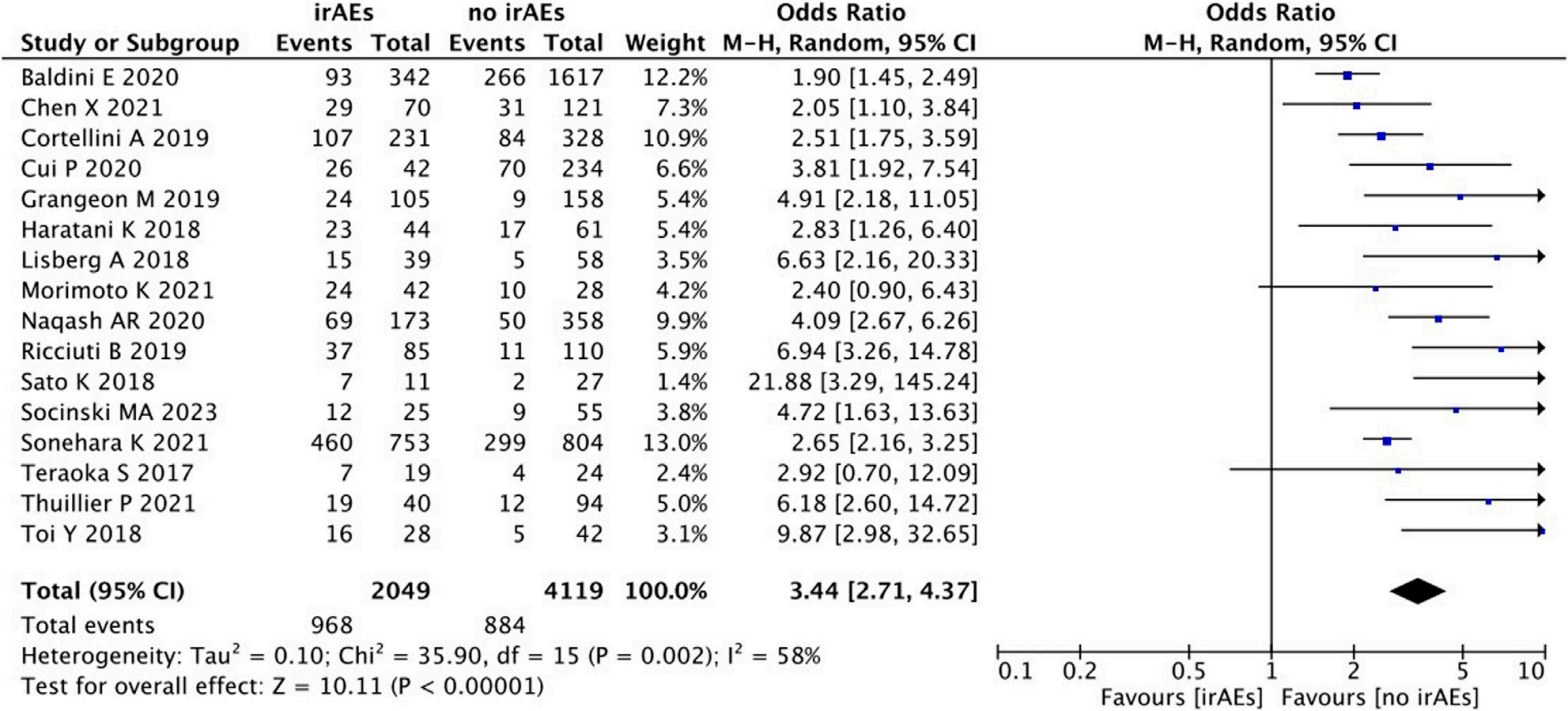
Analysis of ORR between IrAEs and ICI efficacy.

A statistical between-study heterogeneity was found in the OR of the studies (I^2^ = 58%), and a random-effects model was used for merging. As shown in Figure 10, ORR (OR 3.44, 95% CI = 2.71–4.37), *p* < 0.00001) was longer for patients with irAEs than those without irAEs.

## Publication Bias

**FIGURE 11:**
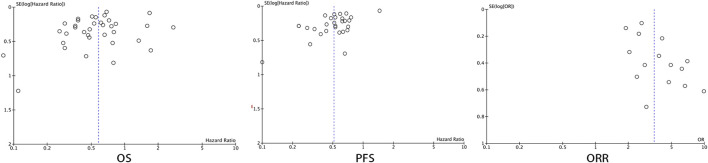
Funnel plot of OS, PFS, and ORR.

Forest plots were used to present the publication bias. The Figure 11 has shown the funnel plots of the OS, PFS, and ORR.

## Discussion

ICIs are believed to initiate irAEs as a consequence of the body’s anti-tumor response (Naidoo et al., 2015; Champiat et al., 2016). Evidence suggests that patients who experienced irAEs are more likely to derive survival benefits from ICIs (Downey et al., 2007; Weber et al., 2012). However, a growing body of evidence has supported this hypothesis. The relationship between irAEs and their impact on clinical efficacy in NSCLC remains poorly defined owing to conflicting data.

In our meta-analysis, we demonstrated significant differences in both PFS/OS and ORR between patients who did and did not experience irAEs, indicating a strong correlation between treatment-related irAEs and their clinical benefits. However, the underlying mechanisms remain unclear. This may be explained by the fact that there may be an abnormal presentation of the molecular mimicry of antigens shared between tumor and normal tissues, which leads to simultaneous T-cell- and B-cell-mediated cross-reactions (Rose and Bona, 1993). This increases the possibility that latent “tissue-specific” autoimmunity may be associated with not only therapy but also healthy tissue. Thus, irAEs are more likely to exacerbate host immune function.

To further evaluate the independent prognostic value of the relationship between treatment-related irAEs and improved clinical outcomes, we performed subgroup analyses to incorporate various clinicopathological covariates. Our results further support the conclusion that this relationship is mediated by confounding factors, such as the number, pathologic subtypes, and various types of irAEs experienced. In our study, no significant difference in OS was found in patients who developed ≥2 irAEs than those who developed <2 irAEs, but PFS showed significant difference. These data suggest that irAEs can serve as a positive predictor of response to therapy, with the balance of its advantages and disadvantages depending on the severity of the irAE itself. In addition, no differences were observed in OS or PFS in terms of pathologic subtypes. This finding indicates that the pathologic subtype had no association with irAEs and survival. IrAEs affecting the skin, gastrointestinal tract, and endocrine system (including thyroid dysfunction) tend to be more manageable than those affecting the lungs and liver. The present study found that patients with these types of irAEs are more likely to experience improved survival benefits. The result may be explained by the toxicities of these irAE subtypes, which could usually be resolved completely with appropriate treatments. Though glucocorticoid is known to be a key element to treat patients with irAEs of pneumonitis and hepatobiliary disease, the effect of steroids still serves as a double-edged sword. The adverse effect of the use of steroids might lead to inferior survival tendency of lung- and liver-related irAEs. In terms of treatment efficacy, further investigation of immune checkpoint therapies based on specific molecular subtypes and genomic alterations may help make informed treatment decisions while maintaining a manageable safety profile.

There are some limitations to our study. First, the retrospective nature and various investigators’ irAE-reporting profiles of all the included studies resulted in an imbalance between the two groups. Additional randomized clinical trials are warranted to address these issues. Second, our study did not include potential confounders, such as the duration of response, discontinuation of therapy, different grades of irAEs, and different ICIs, owing to the limited covariate data available for analysis. Therefore, there is a strong need for high-quality research using additional data to clarify this issue.

In summary, our study validated that patients with NSCLC undergoing ICI treatment may experience irAEs, which may have a persistent response to ICI therapy that can be achieved using irAE as a valuable predictive/prognostic factor. Studies focusing on the molecular mechanisms underlying this correlation may contribute to improved clinical outcomes with ICIs and effective management of their side effects.

## Data Availability

The raw data supporting the conclusion of this article will be made available by the authors, without undue reservation.
